# Long Noncoding RNAs as Biomarkers in Cancer

**DOI:** 10.1155/2017/7243968

**Published:** 2017-05-29

**Authors:** Luka Bolha, Metka Ravnik-Glavač, Damjan Glavač

**Affiliations:** Department of Molecular Genetics, Institute of Pathology, Faculty of Medicine, University of Ljubljana, Ljubljana, Slovenia

## Abstract

Long noncoding RNAs (lncRNAs) are a relatively well-characterized class of noncoding RNA (ncRNA) molecules, involved in the regulation of various cell processes, including transcription, intracellular trafficking, and chromosome remodeling. Their deregulation has been associated with the development and progression of various cancer types, the fact which makes them suitable as biomarkers for cancer diagnosis and prognosis. In recent years, detection of cancer-associated lncRNAs in body fluids of cancer patients has proven itself as an especially valuable method to effectively diagnose cancer. Cancer diagnosis and prognosis employing circulating lncRNAs are preferential when compared to classical biopsies of tumor tissues, especially due to their noninvasiveness, and have great potential for routine usage in clinical practice. Thus, this review focuses on summarizing the perspectives of lncRNAs as biomarkers in cancer, based on evaluating their expression profiles determined in body fluids of cancer patients.

## 1. Introduction

Long noncoding RNAs (lncRNAs) belong to a larger group of noncoding RNAs (ncRNAs) and are generally classified as 200 nt–100 kb long transcripts, lacking the open-reading frame [1, 2]. They are usually transcribed by RNA polymerase II and controlled by the transcriptional activators of the SWI/SNF complex. Most of the generated lncRNA transcripts are usually spliced, capped, and polyadenylated in a similar manner as mRNA molecules [3]. lncRNAs represent a large (>80%) and a very heterogeneous group of ncRNAs, with their expression depending largely on the tissue and cellular context [4–7]. Following the discovery of H19 and XIST lncRNAs in 1990s [8, 9], lncRNA per se was initially regarded as a transcriptional noise with practically no or very little function [10]. However, after being identified as a class of RNA molecules in 2002 [11], studies that followed revealed lncRNA importance and indispensability in various cellular processes, including transcription, intracellular trafficking, and chromosome remodeling [3, 12]. In addition, lncRNAs functioning as regulatory factors have been determined for several complex cellular processes, such as cell death, growth, differentiation, identity establishment; controlling apoptosis, epigenetic regulation, genomic imprinting, alternative splicing, regulation of gene expression at posttranscriptional level, chromatin modification, inflammatory pathologies, and, when deregulated, also in various cancer types [13–23].

**Figure 1:**
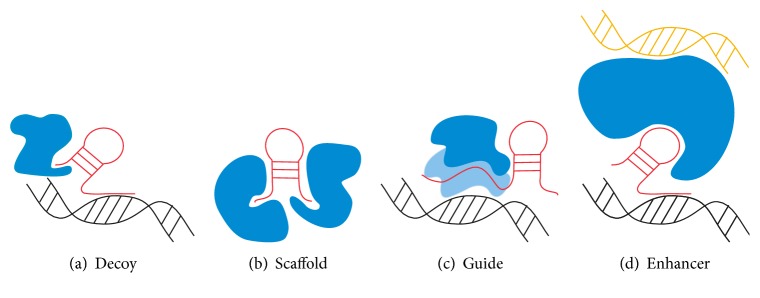
Different mechanisms of long noncoding RNA (lncRNA) action. (a) The lncRNAs can act as decoys, titrating away DNA-binding proteins (e.g., transcription factors); (b) lncRNAs may act as scaffolds to bring two or more proteins to spatial proximity or into a complex; (c) lncRNAs may act as guides to recruit proteins to DNA (e.g., chromatin modification enzymes); (d) lncRNA guidance can also be exerted through chromosome looping in an enhancer-like model in *cis*. lncRNA (red); DNA (black); section of DNA loop (yellow); DNA-binding proteins (blue and light blue). The figure is adapted from John L. Rinn and Howard Y. Chang [12].

lncRNAs can be present in practically all cell compartments [24]. However, many lncRNAs with high abundance were identified especially in the nucleus and cytoplasm [25, 26]. lncRNA secondary structures, such as stem loops and hairpins, results of posttranscriptional modifications, enable their interaction with proteins and chromatin and are crucial for lncRNA's vast set of functions [12]. Some of the main mechanisms of action that allow lncRNAs to have a crucial role in various cellular processes [27] are presented in Figure 1. In general, lncRNAs may act as scaffolds for grouping protein complexes (Figure 1(b)), guides to recruit proteins (Figure 1(c)), transcriptional enhancers by bending chromatin (Figure 1(d)), decoys to release proteins from chromatin (Figure 1(a)), or antagonists for other regulatory ncRNAs, for example, microRNAs (miRNAs) [12, 28].

Regardless of the whole human genome analyses that enabled better understanding of lncRNA expression, function, and distribution in the human genome, classification of lncRNAs remains to be unified [25, 29]. lncRNAs can be sorted according to their structure, sequence, function, localization, metabolism, and interaction with protein-coding genes or other DNA elements [29]. Recently, Wang et al. classified different types of lncRNAs according to their genomic location and context, exerted effect on DNA sequences, mechanism of functioning, and targeting mechanism [30]. In addition, lncRNAs can be classified into several categories including sense lncRNAs, antisense lncRNAs, bidirectional lncRNAs, intronic lncRNAs, intergenic lncRNAs, promoter-associated lncRNAs, and untranslated region- (UTR-) associated lncRNAs [25, 26]. Nevertheless, current classification methods remain inadequate and relatively nontransparent. The general long-term goal is to develop a unified, systematic, and comprehensive lncRNA classification and annotation framework, utilizing global system biology and genomics-driven approaches. Also, the development of improved tools is required for the integration of complex data from multiple types of experiments into this framework, revealing associations between coding and noncoding transcripts. Such lncRNA classification would be a prerequisite for an improved overview and more effective access and usage of large-scale lncRNA data in various fields and applications [29].

Association of lncRNAs in carcinogenesis was observed due to their differential expression in tumors when compared to normal tissues [31]. lncRNAs H19, MALAT1, and PCA3 are highly expressed tumor-associated lncRNAs that were characterized before the availability of next generation sequencing technologies [32–34]. It has been demonstrated that tumorigenesis mostly results from ectopic lncRNA expression [35]. lncRNAs regulate several oncogenes and tumor suppressor genes at transcriptional and posttranscriptional levels, affecting proliferation, apoptosis, angiogenesis, invasion, migration, and metastasis of tumor cells [36–39]. Also, lncRNA-mediated regulation of chromatin remodeling is essential for the integrity of nuclear structure [23]. In recent years, next-generation and high-throughput sequencing techniques have enabled a significant breakthrough in lncRNA identification and characterization. This resulted in continuously rising amounts of data elucidating deregulated lncRNAs associated with the development of various cancer types [40–42]. In this review, we primarily focus on describing circulating lncRNAs present in different body fluids which represent a promising category of biomarkers for cancer diagnosis, prognosis, and also treatment.

## 2. lncRNA-Mediated Epigenetic Modifications

Cancer development and progression can be mediated through multiple mechanisms involving lncRNAs [36, 43–46]. In particular, involvement of lncRNAs has been extensively studied in cancer progression, mainly through epigenetic regulation, activation of oncogenic pathways, and crosstalk with other RNA subtypes [29, 47, 48]. As mentioned before, lncRNAs can interact with chromatin remodeling complexes which usually leads to modifications in the expression of target genes, in either *cis* or *trans* [49]. In these processes, lncRNAs usually recruit chromatin modification factors, for example, DNA methyltransferase enzymes [50], resulting in gene expression variations often inherited within cell lineages [51]. One of the first reported and characterized lncRNA involved in cancer progression through genome-wide epigenetic reprogramming was HOTAIR [52–54]. HOTAIR acts through interaction with polycomb repressive complex 2 (PRC2) subunits, a key chromatin remodeling complex involved in gene silencing [55]. When deregulated, HOTAIR recruits PRC2 subunits in promoter regions of tumor suppressor genes which results in their transcriptional repression and chromatin condensation, thus, favoring tumor progression. Studies have shown that beside HOTAIR, ANRIL, and XIST, lncRNAs also recruit PRC2 in a similar fashion [52, 56, 57].

Studies have shown that over 200 lncRNAs participate in imprinting processes where, depending on their parental origins, specific expression of nearby lncRNAs promotes suppression of neighboring genes in *cis* [58, 59]. Here, instead of acting through, for example, PRC2, lncRNAs recruit DNA methyltransferases directly to modify chromatin conformation and DNA methylation. Among many lncRNAs with such function, several have been characterized, including Kcnq1ot1, TARID, H19, AS1DHRS4, and DACOR1. lncRNAs may also modify nucleosome positioning through SWI/SNF complexes as it was determined for SChLAP1 [60–65]. lncRNA SChLAP1 is overexpressed in a subset of prostate cancers. SChLAP1 can bind directly to hSNF5, one of the core subunits of the SWI/SNF complexes, thus, decreasing their genomic binding. By impairing the proper SWI/SNF regulation of gene expression, SChLAP1 antagonizes tumor suppressive function of the SWI/SNF complexes and promotes tumor cell invasion and metastasis [63, 66]. In addition, NEAT1, UCA1, HIF1A-AS1, and Evf2 also interact with core subunits of SWI/SNF complexes in a similar manner in various cancer types [67]. Other lncRNAs, including Firre, bind chromatin remodelers cohesin and CTCF in order to change the chromatin of whole chromosomes in the process of X chromosome inactivation [68]. lncRNAs may also act as chromatin activators, regulating chromosome looping in their proximity to deposit activating H3K4me3 histone mark on gene promoters [69–71].

## 3. Circulating lncRNAs as Biomarkers in Cancer

Among the main advantages of lncRNAs that make them suitable as cancer diagnostic and prognostic biomarkers is their high stability while circulating in body fluids, especially when included in exosomes or apoptotic bodies [72]. Studies have shown that despite abundant quantities of ribonucleases in different body fluids, lncRNAs were detected in these samples which could successfully resist ribonuclease degradation activities [35]. In addition, lncRNA deregulation in primary tumor tissues is clearly mirrored in various bodily fluids, including whole blood, plasma, urine, saliva, and gastric juice [73–76]. These lncRNA characteristics present an opportunity to develop effective and convenient lncRNA-based biomarkers that are minimally invasive and may be better tolerated by patients, when compared to conventional biopsies, due to their relative noninvasiveness [77]. Detection of circulating cancer-associated lncRNAs in body fluids could be used in the assessment of cancers at distinguishing tumor patients from healthy people at early stages with both high sensitivity and specificity. In addition, predicting the prognosis of tumor patients and the risk of tumor metastasis and recurrence after surgery could be assessed, along with evaluating operation success [35]. Several individual or combined lncRNAs have demonstrated comparable or, in some cases, even higher diagnostic performance than conventional cancer biomarkers, for different cancer types. lncRNA MALAT1 has been identified, by Kaplan-Meier analysis, as an effective prognostic parameter for patient survival in stage I nonsmall cell lung cancer [78]. Also, the measurement of lncRNA PCA3 in patient urine samples has been shown to allow more sensitive and specific diagnosis of prostate cancer than the widely used prostate-specific antigen (PSA) serum levels [79–81]. CEA, CA125, CA153, and AFP are conventional biomarkers, commonly used for breast cancer diagnosis. lncRNA RP11-445H22.4 is overexpressed in breast cancer tissues and can be detected in serum samples, with a sensitivity of 92% and specificity of 74%, which is significantly better than the performance of above listed conventional biomarkers [82]. In addition, diagnostic performances of lncRNAs TINCR, CCAT2, AOC4P, BANCR, LINC00857, AA174084, and H19 were evaluated in body fluid samples (e.g., plasma and gastric juice) of gastric cancer patients. These lncRNAs had the ability to differentiate gastric cancer patients from healthy individuals and to effectively detect different stages of gastric cancer (from early to metastatic cancer forms). However, despite their overall positive diagnostic performances, similar to those obtained by several conventional cancer biomarkers, false-positive and false-negative detections were observed [19, 76, 83]. Also, similar results were obtained after characterizing lncRNAs MALAT1 and PCA3 as biomarkers in prostate cancer patients [84, 85].

Stability of lncRNAs in body fluids of tumor patients has not been thoroughly explored. Studies revealed that some lncRNAs remained stable in plasma under extreme conditions, including several freeze-thawed cycles and prolonged incubation at elevated temperatures [86]. It has also been demonstrated that lncRNAs remained their stability when using plasma and serum from EDTA vacutainer tubes or from tubes lacking the specific anticoagulant, whereas lncRNA amounts declined when using plasma from heparin vacutainer tubes [84].

Three main mechanisms for lncRNA secretion and transport to the extracellular environment have been proposed. First, extracellular RNAs may package themselves into specific membrane vesicles, such as exosomes and microvesicles, in order to be secreted and resist RNase activity. Studies revealed that exosomes most frequently protect plasma lncRNAs [87–90]. Second, extracellular RNAs can be actively released by tumor tissues and cells [84]. However, elevated values of lncRNAs in plasma may have multiple sources, including cancer-adjacent normal cells, immune cells, and other blood cells [86, 90]. Third, extracellular RNAs may encapsulate themselves into high-density lipoprotein (HDL) or apoptotic bodies or are associated with protein complexes, for example, Argonaute- (Ago-) miRNA complex [91] and nucleophosmin 1- (NPM1-) miRNA complex [92]. However, despite many performed studies, secretion and transport mechanisms of lncRNAs to the circulation system remain poorly understood, mostly because several studies tend to contradict each other. Also, thorough examinations and reports regarding biological functions of lncRNAs in cancers are still lacking [35].

In order to introduce circulating lncRNAs into clinical practice, further studies and improvements should be performed regarding the standardization of sample preparation protocols, endogenous controls of lncRNAs in body fluids and the extraction methods should be uniformed, standards assessing the quality of lncRNAs and the credibility of qPCR results should be more accurate and reliable, and more high-quality research studies should be performed, with selection bias reduced as much as possible [35]. In addition, several technical obstacles remain to be addressed and overcome in the future, to enable a reliable use of circulating lncRNAs as effective cancer biomarkers. Commercial kits employing columns are mostly used for lncRNA extraction from body fluids. Unfortunately, no consistent results have been obtained regarding the differences in the efficiency of column-based methods, indicating that comparison and standardization of lncRNA extraction methods are necessary [93]. Absolute concentration of lncRNAs in body fluids is usually low and frequently requires an RNA amplification step prior analysis, which is time consuming and can be problematic when results are needed promptly [94]. It has also been observed that RNA extracted from plasma and serum samples may be undetectable when using a NanoDrop spectrophotometer for quantifying circulating RNAs [93]. This makes the necessity for the development of highly sensitive methods for quantifying lncRNAs crucial. Also, since the mechanisms of lncRNA secretion are not yet fully understood, the levels of circulating lncRNAs may be affected by other concomitant disease changes, besides tumorigenesis. Thus, overrated amounts of specific lncRNAs associated with a particular disease may be determined [94]. There are also several existing obstacles regarding the techniques, commonly used for quantifying circulating lncRNAs. Quantitative RT-PCR is a well-established method for detecting and quantifying circulating RNAs. However, the cost per sample is relatively high and the throughput of the method low [93]. Recently developed assays, such as the Human Disease-Related lncRNA Profiler (System Biosciences SBI), allow the measurement of a panel of lncRNAs but can detect only annotated lncRNAs. Therefore, only a medium throughput can be attained [93]. Commercial lncRNA microarray platforms can be used to detect only previously described biomarkers already present in the lncRNA databases. Microarrays have a high throughput, but a lower dynamic range and specificity, when compared to qRT-PCR and RNA-seq [93]. RNA-seq can be used for the identification of known and new lncRNA species, with lower cost per sample than microarrays and qRT-PCR. However, a relatively large amount of starting material is required (cca. 1 *μ*g RNA), which is difficult to extract from biological fluids, for example, plasma or serum samples. In addition, current RNA-seq methodology is expensive and complex and requires a special equipment with a trained personnel [93].

Since expression profiles of cancer-associated lncRNAs may be very specific for various cancer types, these specific lncRNAs could be efficiently used as tumor biomarkers in different body fluids in the near future, with vital significance for clinical research [35]. In the following section, we describe some of these lncRNAs.

## 4. lncRNAs as Cancer Biomarkers Obtained from Body Fluids

Deregulated expression of lncRNAs is strongly linked to the development of various tumors and can be relatively effectively detected in patient's body fluids for several cancer types [77]. Regarding their involvement in malignant disease development, when comparing to normal tissues of healthy individuals, lncRNAs are generally divided into oncogenic or tumor suppressive, being upregulated or downregulated, respectively [31, 45]. Sets of a number of differentially expressed cancer-associated lncRNAs in a variety of cancers are presented in Tables 1 and 2. Among them, several lncRNAs represent promising noninvasive cancer biomarkers for detection in patient's body fluids, including PCA3, HOTAIR, HULC, MALAT1, H19, LINC00152, RP11-160H22.5, XLOC_014172, LOC149086, AA174084, and UCA1. Moreover, for several of these lncRNAs, it has been already demonstrated that they could be effectively used as diagnostic and prognostic cancer biomarkers in clinical practice.

PCA3 has been recently approved as a urine biomarker for prostate cancer by the US Food and Drug Administration [73]. This lncRNA allows better sensitivity and specificity when compared to the widely used PSA blood test, mainly because of its significantly higher expression in prostate cancer patients [79–81, 95–97]. A meta-analysis of several studies has determined the validity of PCA3 levels in urine samples for prostate cancer diagnosis, with a summary sensitivity of 62% and specificity of 75%. In the receiver operating characteristic (ROC) curve analysis, this translated to an area under the ROC curve (AUC) of 0.75 [98]. PCA3 has also a prognostic value for prostate cancer, since its expression levels correlate well with tumor aggressiveness [99, 100].

HOTAIR was found to be highly expressed in saliva samples of oral squamous cell carcinoma (OSCC) patients. Since higher expression levels of HOTAIR were determined for metastatic patients, this lncRNA represents a strong candidate for metastatic oral cancer diagnosis [74]. In addition, the association between increased blood levels of HOTAIR and poor prognosis with higher mortality in colorectal cancer patients has been determined. Expression levels of HOTAIR could also predict the survival time of patients. Evaluated diagnostic performance of HOTAIR in peripheral blood cells has shown its sensitivity of 67%, specificity of 92.5%, and AUC of 0.87. Thus, HOTAIR represents an effective negative prognostic biomarker for colorectal cancer in blood samples [101].

HULC can be effectively detected in plasma and peripheral blood cells and is significantly overexpressed in hepatocellular carcinoma patients, thus, representing a prominent novel biomarker for liver cancer. However, no data regarding HULC diagnostic performance are available at this time [102, 103]. HULC detected in blood has also been recently proposed as a diagnostic biomarker for gastric cancer [104].

MALAT1 represents a promising diagnostic biomarker detectable in blood, to effectively screen lung cancer. One study has shown downregulation of MALAT1 in blood samples of lung cancer patients which was contrary to MALAT1 levels in lung cancer tissues, where it was significantly upregulated. Conversely, MALAT1 showed elevated expression levels in whole blood of metastatic lung cancer patients [105]. Due to its relatively low expression and low detection sensitivity (sensitivity 56%; specificity 96%; AUC 0.79) in diagnosis of non-small-cell lung cancer (NSCLC), MALAT1 is not regarded suitable as an independent biomarker to diagnose lung cancer but should be rather used as a complementary biomarker [106]. In addition to lung cancer, MALAT1 has proven itself as a prominent biomarker with its elevated expression detected in plasma and urine of prostate cancer patients, with a sensitivity and specificity of 58.6% and 84.8%, respectively (AUC 0.836). MALAT1 also helped to predict the outcome of prostate biopsies [84, 107].

Elevated expression profiles of H19 have been determined in plasma samples of gastric cancer patients. H19 has great potential as a promising biomarker due to its high diagnostic value for the detection of gastric cancer (sensitivity 82.9%; specificity 72.9%; AUC 0.838). It has also been more effective in early stage gastric cancer diagnosis than the conventional biomarkers, such as CEA and CA199, with a sensitivity of 85.5%, specificity of 80.1%, and AUC of 0.877 [83].

Expression levels of LINC00152 in plasma were found to be significantly increased in early and advanced gastric cancer patients. This lncRNA had also significantly higher expression profiles in postoperative plasma samples. The diagnostic value of LINC00152 (sensitivity 48.1%; specificity 85.2%; AUC 0.675) was better than those of CEA and CA199 biomarkers, which makes LINC00152 a good candidate as a novel blood-based biomarker for gastric cancer diagnosis [90]. In addition, LINC00152 could also be detected in the gastric juice of patients with gastric cancer [108].

Among the less commonly studied lncRNAs belong RP11-160H22.5, XLOC_014172, and LOC149086 which have been proposed as biomarkers for the diagnosis of hepatocellular carcinoma in patient plasma samples. These three lncRNAs had better scores for hepatocellular carcinoma diagnosis when used in combination, in comparison to each individual lncRNA, with a merged AUC of 0.896, sensitivity of 82%, and specificity of 73% [109]. In addition, XLOC_014172 and LOC149086 lncRNAs had also a good prognostic value for metastasis prediction (sensitivity 91%; specificity 90%; AUC for the combined lncRNAs 0.675) [109].

AA174084 represents a relatively robust but specific biomarker, suitable for the diagnosis of gastric cancer in gastric juice samples (sensitivity 46%; specificity 93%; AUC 0.848). Levels of AA174084 in patient's gastric juices were found to be significantly upregulated when compared to those of healthy individuals. Interestingly, this lncRNA was not suitable for the diagnosis of gastric cancer from plasma samples [76].

UCA1 lncRNA has been identified as a potential biomarker for bladder cancer. Due to its relatively high overall specificity, it has a high potential to discriminate between the bladder/urothelial cancer and other cancer types, or other diseases related to the urinary tract (sensitivity 80.9%; specificity 91.8%; AUC 0.882). UCA1 can be detected in urine samples of bladder cancer patients, mostly in the cellular sediments [110].

Additional, continuously increasing amounts of information regarding cancer-associated lncRNAs, including those detected in body fluids, can be obtained from many existing databases, several of which are presented in Table 3.

## 5. Conclusions and Perspectives

lncRNAs represent a relatively large and heterogeneous group of ncRNAs and are considered as suitable diagnostic and prognostic biomarkers in cancer. In recent years, circulating lncRNAs have proven themselves extremely valuable for the detection of various cancer types. Their usage as biomarkers is convenient not only because samples containing circulating lncRNAs can be easily and noninvasively obtained from cancer patients but also because these lncRNAs remain relatively stable in body fluids. They can be quite easily detected in whole blood, plasma, serum, urine, saliva, and gastric juice samples, by using a variety of common molecular biology techniques, such as qRT-PCR, microarray hybridization, and sequencing (e.g., RNA-seq). Because lncRNAs are usually differentially abundant in different body fluids, mainly depending on the cancer type, effective cancer diagnosis and prognosis currently depend on combining different candidate lncRNAs, together with previously established biomarkers. Some circulating lncRNAs have already been proven as promising and sensitive biomarkers, and there are likely more to come.

## Figures and Tables

**Table 1:** Upregulated cancer-associated lncRNAs when compared to normal tissues.

Name	Cancer type	Fold change^a^	References
Wt1-as	Acute myeloid leukemia	NA^b^	[111]
XIST	Glioma	NA	[112]
CRNDE	Glioma	NA	[113]
MALAT1	Glioma	2.0–5.0	[114]
	Colorectal	2.0–6.0	[115]
	Lung	>40.0	[116]
	Prostate	NA	[84]
	Hepatocellular	NA	[117]
	Uterus	NA	[118]
LSINCT5	Breast	2.0–7.0	[119]
LINC00617	Breast	>1.5	[120]
RP11-445H22.4	Breast	15.0–20.0	[121]
BC200	Breast	NA	[122]
CCHE1	Cervical	NA	[123]
CCAT1-L	Colorectal	NA	[71]
POU3F3	Esophageal	NA	[124]
PCAT-1	Colorectal	NA	[125]
	Prostate	NA	[126]
HOTAIR	Esophageal	NA	[127]
	Lung	NA	[128]
	Cervical	NA	[129]
	Pancreas	NA	[130]
	Breast	NA	[52]
	Oral	NA	[131]
	Hepatocellular	>2.0	[132]
	Glioma	NA	[133, 134]
	Colorectal	5.2	[101]
CCAT2	Lung	7.5	[135]
	Colon	NA	[136]
	Cervical	NA	[137]
LINC00152	Gastric	NA	[35, 90]
LSINCT-5	Gastric	NA	[138]
HOXA11-AS	Glioma	NA	[139]
Linc-POU3F3	Glioma	>2.6	[140]
ATB	Glioma	5.0–10.0	[141]
AB073614	Glioma	NA	[142]
RP11-160H22.5	Hepatocellular	2.5	[109]
XLOC_014172	Hepatocellular	67.7	[109]
LOC149086	Hepatocellular	4.6	[109]
BANCR	Hepatocellular	NA	[143]
SNHG3	Hepatocellular	NA	[144]
MVIH	Hepatocellular	3.75	[145]
	Lung	NA	[146]
LCAL1	Lung	NA	[147]
LUADT1	Lung	NA	[148]
AFAP1-AS1	Lung	NA	[149]
	Colorectal	NA	[150]
	Hepatocellular	NA	[151]
	Esophageal	>1.0	[152]
ANRIL	Lung	>1.5	[153]
	Hepatocellular	>1.0	[154]
	Bladder	>1.0	[155]
UCA1	Lung	NA	[156]
	Oral	NA	[157]
	Bladder	32.9	[158]
	Colon	NA	[159]
	Hepatocellular	NA	[160]
	Breast	NA	[161]
	Esophageal	>2.0	[162]
CASC15	Melanoma	NA	[163]
SPRY4-IT1	Melanoma	>2.0	[164, 165]
	Glioma	NA	[166]
H19	Bladder	NA	[167]
	Gastric	NA	[83]
	Esophageal	NA	[168]
	Colorectal	NA	[169]
	Glioma	NA	[170]
HULC	Pancreas	NA	[171]
	Hepatocellular	32.7	[102]
	Glioma	NA	[172]
PCA3	Prostate	NA	[34, 99]
PCAT5	Prostate	NA	[173]
PCAT18	Prostate	8.8–11.1	[174]
PRNCR1	Prostate	NA	[175]
NEAT1	Glioma	NA	[176, 177]
	Oral	NA	[74]
	Hepatocellular	NA	[178]
	Nasopharyngeal	NA	[179]
PVT1	Thyroid	NA	[180]
	Gastric	NA	[181]
	Colorectal	NA	[182]
SRA	Breast	NA	[183]

^a^Fold change values, relative to normal controls; ^b^not available (data is presented in a graphical format in the original report).

**Table 2:** Downregulated cancer-associated lncRNAs when compared to normal tissues.

Name	Cancer type	Fold change^a^	References
MEG3	Glioma	NA^b^	[184, 185]
ZFAS1	Breast	2.0	[186]
GAS5	Breast	<1.0	[187]
	Glioma	NA	[188]
LOC554202	Colorectal	NA	[189]
CUDR	Gastric	NA	[190]
PTENP1	Gastric	NA	[191]
	Prostate	NA	[191]
AA174084	Gastric	3.2	[76]
LINC00982	Gastric	7.7	[192]
TSLC1-AS1	Glioma	NA	[193]
ADAMTS9-AS2	Glioma	NA	[194]
MDC1-AS	Glioma	NA	[195]
TUG1	Glioma	NA	[196]
ROR	Glioma	NA	[197]
CACS2	Glioma	NA	[198]
PRAL	Hepatocellular	NA	[199]
MALAT1	Lung	3.3	[106]
AK023948	Papillary thyroid carcinoma	5.0	[200]

^a^Fold change values, relative to normal controls; ^b^not available (data is presented in a graphical format in the original report).

**Table 3:** Databases containing lncRNA data.

Name	URL	References
CHIPbase	http://deepbase.sysu.edu.cn/chipbase/	[201]
C-It-Loci	http://c-it-loci.uni-frankfurt.de/	[202]
Co-LncRNA	http://www.bio-bigdata.com/Co-LncRNA/	[203]
DIANA-LncBase	http://www.microrna.gr/LncBase	[204, 205]
Linc2GO	http://www.bioinfo.tsinghua.edu.cn/~liuke/Linc2GO/index.html	[206]
Lnc2Cancer	http://www.bio-bigdata.com/lnc2cancer/	[207]
LncACTdb	http://www.bio-bigdata.net/LncACTdb/	[208]
LNCipedia	http://www.lncipedia.org/	[209, 210]
LncRBase	http://bicresources.jcbose.ac.in/zhumur/lncrbase/	[211]
LncRNA2Function	http://mlg.hit.edu.cn/lncrna2function/	[212]
LncRNAdb	http://www.lncrnadb.org/	[213, 214]
LncRNADisease	http://210.73.221.6/lncrnadisease	[215]
lncRNASNP	http://bioinfo.life.hust.edu.cn/lncRNASNP/	[216]
LncRNome	http://genome.igib.res.in/lncRNome/	[217]
miRcode	http://www.mircode.org	[218]
NONCODE	http://www.noncode.org/	[219, 220]
Starbase 2.0v	http://starbase.sysu.edu.cn/rbpLncRNA.php	[221, 222]
